# The Emerging Role of Endothelial Ion Channels in the Control of Human Microcirculation

**DOI:** 10.3390/ijms27031421

**Published:** 2026-01-30

**Authors:** Francesco Moccia, Valentina Brunetti, Roberto Berra-Romani, Giovanni Villone, Gennaro Raimo, Teresa Soda, Giorgia Scarpellino, Germano Guerra

**Affiliations:** 1 Department of Medicine and Health Sciences “V. Tiberio”, University of Molise, 86100 Campobasso, Italy; giovanni.villone@unimol.it (G.V.); raimo@unimol.it (G.R.); germano.guerra@unimol.it (G.G.); 2 Department of Biology and Biotechnology “L. Spallanzani”, University of Pavia, 27100 Pavia, Italy; valentina.brunetti01@universitadipavia.it (V.B.); giorgia.scarpellino@unipv.it (G.S.); 3 Department of Biomedicine, School of Medicine, Benemérita Universidad Autónoma de Puebla, Puebla 72410, Mexico; rberra001@hotmail.com; 4 Department of Health Sciences, University of Magna Graecia, 88100 Catanzaro, Italy; teresa.soda@unicz.it

**Keywords:** vascular tone, endothelial cells, inward rectifier K^+^ channel, small- and intermediate-conductance Ca^2+^-activated K^+^ channels, ATP-dependent K^+^ channels, nitric oxide, Ca^2+^ signaling, TRP channels

## Abstract

Endothelial ion signaling is crucial for the proper function of the arterial microcirculation, regulating local blood flow to meet metabolic demands and contributing to the regulation of systemic arterial pressure. The role of endothelial ion channels in the precise control of vascular resistance has been primarily investigated in animal models, where the microvasculature is more readily accessible. This review aims to discuss current knowledge on the role of endothelial ion signaling in vasomotor regulation in the human microcirculation, focusing on potassium (K^+^) channels (K_IR_2.1, K_ATP_, SK_Ca_/IK_Ca_), Transient Receptor Potential (TRP) channels, particularly TRP Vanilloid 1 (TRPV1) and TRPV4, and Piezo1 channels. The analysis examines the organization of the endothelial ionic signaling machinery in the most extensively studied human microvascular beds, such as the skin, skeletal muscle, and brain, while also discussing vascular reactivity in vessels isolated ex vivo. Accumulating evidence indicates that a distinct repertoire of endothelial ion channels engages diverse endothelium-dependent vasorelaxant pathways across different vascular beds. Understanding how endothelial channels regulate the microvascular unit is predicted to foster the search for alternative therapeutic strategies for treating cardiovascular and neurodegenerative disorders associated with endothelial dysfunction.

## 1. Introduction

**Figure 1:**
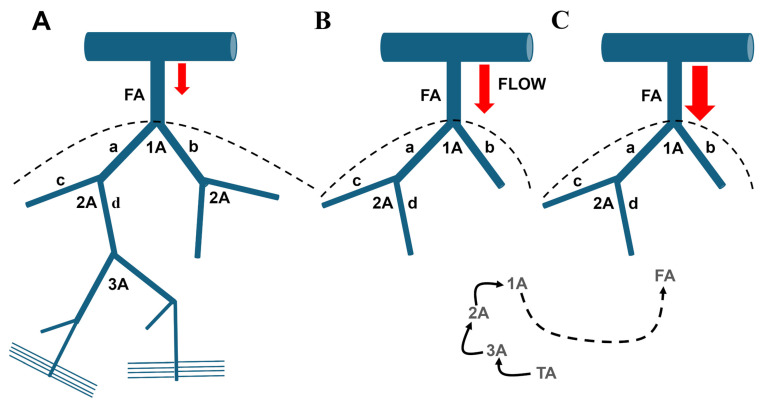
Schematic depiction of the microvascular unit. (**A**), the illustration shows a feed artery (FA) originating from a large conduit artery. The FA delivers blood to the tissue (e.g., skin and skeletal muscle; dotted outline) and gives rise to two primary (1A) arterioles (labeled as a and b). Each 1A arteriole branches into second-order (2A) arterioles, which further divide into third-order (3A) and ultimately terminal (TA) arterioles, with each TA supplying clusters of capillaries (postcapillary microvessels are not depicted for clarity). (**B**), vasodilation initiated in 3A and 2A arterioles (branches c and d) propagates retrogradely (“ascends”) into the parent 1A arteriole (branch a) but does not extend into the proximal FA. (**C**), when dilation of the daughter arterioles supplying branch b is enhanced, vasodilation ascends into branch b and, together with signals conducted along branch a, is integrated within the FA. Dilation of the FA decreases proximal vascular resistance that otherwise restricts tissue perfusion. As a result, total blood flow into the microcirculation increases markedly.

Endothelial signaling is essential for the primary function of arterial microcirculation, ensuring that local blood flow is sufficient to meet the metabolic demands of parenchymal cells and regulating systemic blood pressure [1,2,3]. The microcirculation typically consists of a network of small resistance arteries and arterioles that branch off from larger feed arteries, which have internal diameters of up to 300–400 μm. As these vessels branch, they gradually decrease in size, with arterioles, known as terminal arterioles, reaching internal diameters between 10–20 μm (Figure 1). The microcirculation is the primary site of vascular peripheral resistance, being the location where the mean arterial pressure (MAP) drops from ~65–80 mmHg (feed artery at the inlet of the terminal arteriole) to ~20–30 mmHg (capillaries at the outlet of the terminal arteriole) [1,2,4]. The functional unit of the microcirculation, which delivers oxygen and nutrients to parenchymal cells, comprises a terminal arteriole which supplies blood to 10–20 capillaries under the control of a pre-capillary sphincter. The innermost layer of microvessels is composed of a continuous sheet of endothelial cells that physically interact with diverse types of mural cells in different segments of the vascular tree [1,2,4]. In the terminal arteriole, the endothelial monolayer is encased by a single layer of smooth muscle cells that encircle the endothelial tube. The number of smooth muscle cell layers increases as the arterioles transition into small resistance arteries and then large conduit arteries. The abluminal side of the first few capillaries branching off from the terminal arteriole is covered by contractile pericytes, while more distal capillaries are coated with non-contractile pericytes that cover a lower percentage of the endothelial surface. Resistance arteries and arterioles can develop high contractile forces in response to an increase in luminal pressure, a mechanism known as autoregulation. Additionally, these vessels may dynamically change their diameter, thereby regulating both MAP (vasodilation: MAP decreases; vasoconstriction: MAP increases) and blood supply to downstream capillaries [1,2,4]. The fine-tuned control of capillary perfusion is achieved through contraction or relaxation of ensheathing pericytes at the arteriole-to-capillary transition (ACT) zone [5].

The vasomotor activity of vascular smooth muscle cells (VSMCs) and contractile pericytes is regulated by spatiotemporally regulated changes in the resting membrane potential (V_M_) and in the intracellular Ca^2+^ concentration ([Ca^2+^]_i_) [1,3,4,6]. Nevertheless, the endothelial ion signaling machinery is critical for the modulation of both electrical and Ca^2+^ signals in mural cells and for coordinating the transmission of vasorelaxant signals from the active site to upstream vessels [1,4,7]. The endothelial monolayer serves as a signal transduction platform that integrates physical and chemical cues derived from parenchymal cells and flowing blood by generating vasoactive mediators, such as nitric oxide (NO), prostaglandin I2 (or prostacyclin), and endothelium-dependent hyperpolarization (EDH), which elicit arteriolar dilation and redirect blood flow from inactive to metabolically active areas (Figure 1) [1,3,4,6,8]. A plethora of studies conducted in animal models of microcirculation has revealed that multiple endothelial ion channels, e.g., inwardly rectifying K^+^ channels, ATP-dependent K^+^ channels, small- and intermediate-conductance Ca^2+^-activated K^+^ channels (SK_Ca_/IK_Ca_), Transient Receptor Potential (TRP) channels, and Piezo1 channels, are key regulators of MAP and capillary perfusion, as widely discussed elsewhere [1,3,4,7,9,10,11].

Herein, we discuss the role of endothelial ion channels in the fine-tuning regulation of vasoactive behavior in the human microcirculation. We first describe the biophysical features of the most relevant ion channels involved in the regulation of human microcirculation, namely several K^+^ channels, some TRP isoforms, and Piezo1 channels. Then, we discuss how the endothelial ion signaling machinery is organized to regulate peripheral vascular resistance and regulate blood flow to downstream capillaries in the most investigated human microvascular beds, including the forearm and cutaneous microcirculations. We also discuss the putative role of endothelial ion signaling in the regulation of cerebral blood flow (CBF). Finally, we illustrate the role of endothelial ion channels in the regulation of vascular tone in ex vivo human vessels, i.e., coronary and adipose arterioles.

## 2. Endothelial Ion Channels and Vasomotor Control in Animal Studies and Cultured Endothelial Cells

In contrast to murine models, information regarding the role of endothelial ion channels in regulating smooth muscle cell contractility within the human microvasculature remains limited. Vascular endothelial cells can control vascular tone through electrical and Ca^2+^ signals, which may act locally or be retrogradely propagated to upstream vessels (electrical signals propagate faster than intercellular Ca^2+^ waves). Herein, we focus on four distinct endothelial signaling pathways that are critical for the vasomotor control of the mouse/rat microcirculation through the modulation of the resting membrane potential (V_M_) and the resting intracellular Ca^2+^ concentration ([Ca^2+^]_i_): (1) EDH; (2) InsP_3_Rs and SOCE; (3) TRP channels; and (4) Piezo1 channels. Animal studies have revealed that EDH is crucial for regulating the diameter of smaller resistance-sized arteries and resistance arterioles, while NO, as well as prostaglandin I2 and prostacyclin, promote vasorelaxation of large conduit arteries in the most widely employed animal models of systemic circulation, i.e., rat, mouse, hamster, guinea-pig and pig [3,12,13]. This section provides the conceptual framework for understanding the rationale behind investigating specific vasorelaxing signaling pathways in the human microcirculation (see Section 3). Accordingly, the evidence discussed herein is derived exclusively from animal studies, predominantly performed in rats and mice.

### 2.1. An Increase in [Ca^2+^]_i_ Is Critical for Initiating Endothelium-Dependent Vasorelaxation

**Figure 2:**
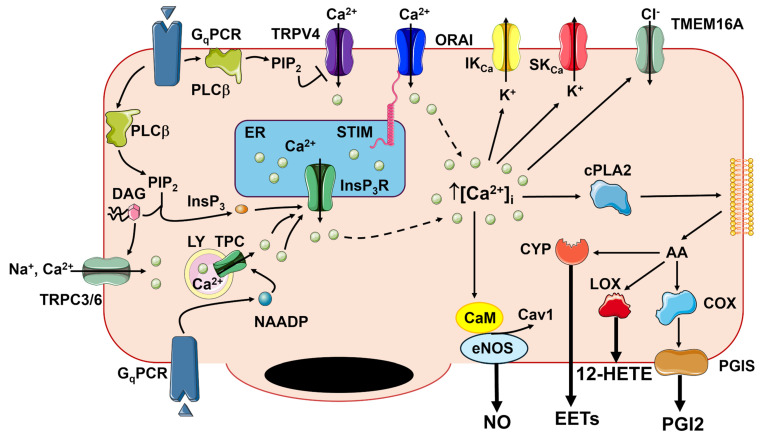
The role of endothelial Ca^2+^ signals in vasodilation. Extracellular agonists, such as acetylcholine, ATP, histamine, bradykinin, and glutamate, bind to their specific G_q_ protein-coupled receptors (G_q_PCRs), thereby stimulating phospholipase Cβ (PLCβ) to cleave phosphatidylinositol 4,5-bisphosphate (PIP_2_) into diacylglycerol (DAG) and 1,4,5-inositol-trisphosphate (InsP_3_). DAG directly activates extracellular Ca^2+^ entry through TRP Canonical 3 and 6 (TRPC3 and TRPC6), while InsP_3_ elicits Ca^2+^ release from the endoplasmic reticulum (ER) through InsP_3_ receptors (InsP_3_Rs). Additionally, the hydrolysis of PIP_2_ relieves the PIP_2_-dependent inhibition of TRPV4, which can also contribute to Ca^2+^ influx. InsP_3_-dependent ER Ca^2+^ mobilization also requires lysosomal Ca^2+^ release through two pore channels (TPCs), which are gated by nicotinic acid adenine dinucleotide phosphate (NAADP). The mechanisms leading to NAADP synthesis in vascular endothelial cells are poorly understood, but NAADP production is through to be mediated from the precursor NAD(P) by (dual) NADPH oxidases (NOX/DUOX) enzymes, such as NOX5, DUOX1, and DUOX2. The resulting decrease in ER Ca^2+^ concentration activates the ER Ca^2+^ sensor, STIM, which in turn binds to and gates the plasma membrane Ca^2+^-permeable channel Orai, thereby leading to store-operated Ca^2+^ entry (SOCE). These signaling pathways contribute to elevating the intracellular Ca^2+^ concentration ([Ca^2+^]_i_) in vascular endothelial cells. Endothelial Ca^2+^ signals recruit different Ca^2+^-sensitive effectors to promote vasodilation. Cytosolic Ca^2+^ displaces caveolin 1 (Cav1) from endothelial nitric oxide (NO) synthase (eNOS), thereby removing the tonic inhibition and triggering NO release. Furthermore, Ca^2+^ activates cytosolic phospholipase A2 (cPLA2) to cleave arachidonic acid (AA) from membrane phospholipids. Subsequently, AA is converted into the following vasorelaxant mediators: (1) prostaglandin (PGI_2_) by the combined action of cyclooxygenases (COX-1 and COX-2) and PGI_2_ synthase (PGIS); (2) 12-HETE (12(*S*)-hydroxyeicosatrienoic acid), 15-HETE, 11,12,15-THETA (11,12,15-trihydroxyeicosatrienoic acid) by various lipoxygenases (LOX; e.g., 5-LOX, 12/15-LOX, 12-LOX, and 15-LOX); and (3) epoxyeicosatrienoic acids (EETs) by CYP epoxigenases 2 C8/9 (CYP). Finally, endothelial Ca^2+^ signals promote endothelial hyperpolarization by activating the small- and intermediate-conductance Ca^2+^-activated K^+^ channels (SK_Ca_/IK_Ca_) channels and the Ca^2+^-activated Cl^−^ channel TMEM16A. This schematic is based upon the following references: [1,3,6,19,34,35,36,37,38,39,40,41].

Vascular endothelial cells express a wide array of G_q_PCRs that enable them to sense both blood-borne and parenchymal autacoids, including acetylcholine, adenosine trisphosphate (ATP), bradykinin, histamine, thrombin, and glutamate [1,2,3,7,8,11,14,15,16,17,18,19,20,21,22,23,24,25]. The Ca^2+^ response to the endothelial autacoids is initiated by inositol-1,4,5-trisphosphate (InsP_3_)-dependent Ca^2+^ release from the endoplasmic reticulum (ER), which manifests as local Ca^2+^ release events, regenerative Ca^2+^ transients, or intercellular Ca^2+^ waves (Figure 2) [3]. Recent studies have revealed that lysosomal Ca^2+^ release through Two-Pore Channels (TPCs) (Figure 2) or TRP Mucolipin 1 (TRPML1) channels can further sustain intracellular Ca^2+^ signaling either by triggering Ca^2+^-induced Ca^2+^ release (CICR) through InsP_3_Rs (Figure 2) or by reloading the ER Ca^2+^ pool [26,27,28]. In contrast, naïve endothelial cells lack ryanodine receptors [7]. InsP_3_-induced ER Ca^2+^ mobilization causes a dramatic reduction in the ER Ca^2+^ concentration ([Ca^2+^]_ER_), which is sensed by STIM proteins. STIM1 and STIM2 are two single-pass transmembrane proteins that are activated by depletion of the [Ca^2+^]_ER_; upon activation, they oligomerize and migrate to ER-plasma membrane junctions (*puncta*), where they tether and gate the Ca^2+^-selective channel, Orai (Figure 2) [29,30,31]. In vascular endothelial cells, STIM2 is primarily responsible for constitutive Ca^2+^ entry, while STIM1 mediates agonist-induced SOCE activation. Of the three known Orai isoforms (Orai1-3), Orai1 is the predominant contributor to the endothelial SOCE [19,32,33]. Endothelial Ca^2+^ signaling stimulates SK_Ca_/IK_Ca_ channels to mediate EDH (Figure 2) and promotes the production of NO and PGI2, and additional vasorelaxant mediators derived from arachidonic acid (AA) metabolism, such as epoxyeicosatrienoic acids (EETs), 12-HETE (12(*S*)-hydroxyeicosatrienoic acid), 15-HETE, 11,12,15-THETA (11,12,15-trihydroxyeicosatrienoic acid) (Figure 2).

### 2.2. Endothelium-Dependent Hyperpolarization (EDH)

**Figure 3:**
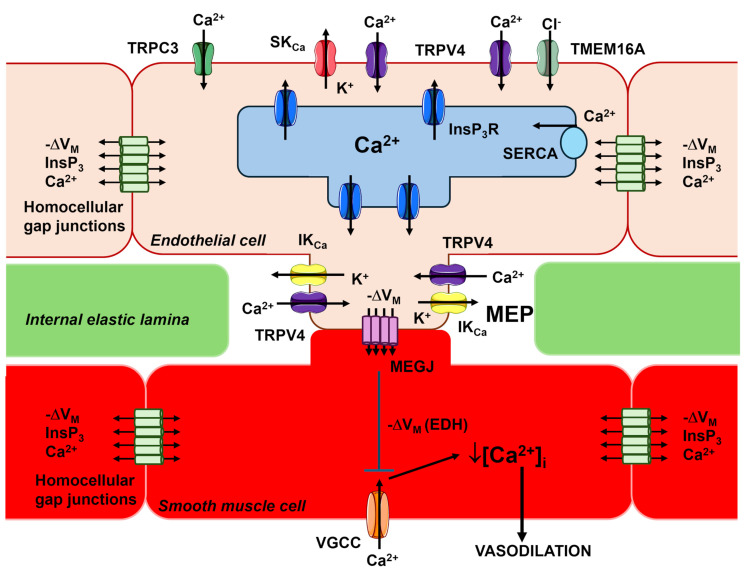
Endothelial ion signaling at the myo-endothelial projection (MEP). Myoendothelial projections (MEPs) are endothelial cell extensions that traverse the internal elastic lamina towards an adjacent vascular smooth muscle cell (VSMC). Additionally, homocellular gap junctions allow the bidirectional transfer of electrical and chemical signals between adjacent endothelial cells and VSMCs. MEPs represent a specialized signaling microdomain site in which myo-endothelial gap junction (MEGJs) enable the transfer of electrical (e.g., hyperpolarization, −ΔV_M_) and chemical (e.g., InsP_3_ and/or Ca^2+^) signals. MEPs are structurally and functionally more prominent within the walls of peripheral resistance arterioles than in conduit arteries. At MEPs, Transient Receptor Potential Vanilloid family member 4 (TRPV4), intermediate-conductance Ca^2+^-activated K^+^ (IK_Ca_) channels, and inositol 1,4,5-trisphosphate receptors (InsP_3_Rs) are spatially organized into signaling complexes that elicit endothelial cell hyperpolarization (−ΔV_M_) in response to vasodilator agonists [6,52,53]. The signaling pathway whereby G_q_PCRs activate InsP_3_Rs and TRPV4 channels has been illustrated in Figure 1. IK_Ca_ channels cluster at MEPs, while SK_Ca_ channels are primarily located at the cell periphery. ER Ca^2+^ release through InsP_3_Rs and extracellular Ca^2+^ entry through TRPV4 channels provide the primary source of Ca^2+^ for the recruitment of SK_Ca_/IK_Ca_ channels. Endothelial-dependent hyperpolarization (−ΔV_M_) is then transmitted to overlying VSMC through MEGJs, thereby reducing the open probability of voltage-gated L-type Ca^2+^ channels and inducing relaxation. Endothelial-dependent hyperpolarization can also be contributed by the Ca^2+^-activated Cl^−^ channel, TMEM16A, while TRPC3 provides an additional pathway for Ca^2+^ entry [6,40,52,53,54,55].

The resting V_M_ of microvascular endothelial cells in intact pressurized mouse microvessels ranges between −35 and −40 mV [40,42,43] and is highly sensitive to an increase in K^+^ permeability, which can shift V_M_ towards the K^+^ equilibrium potential (~−85 mV) [44]. According to the current model, EDH represents an integrated endothelial signaling mechanism driven by distinct K^+^ channels operating in arterioles and capillaries. In arterioles, including the ACT zone [45], agonist-induced endothelial Ca^2+^ signals activate SK_Ca_ (K_Ca_2.3) and IK_Ca_ (K_Ca_3.1) channels, which are abundantly expressed in arteriolar endothelial cells [3,4,7,44,46,47,48,49]. While endothelial IK_Ca_ channels cluster at MEPs, SK_Ca_ channels are primarily located at the cell periphery [6]. Their activation generates endothelial hyperpolarization that propagates to overlying smooth muscle cells and pericytes through myo-endothelial projections (MEPs) containing myo-endothelial gap junctions (MEGJs), leading to the closure of voltage-gated L-type Ca^2+^ channels and subsequent vasorelaxation (Figure 3) [1,4,6,12,49]. EDH is propagated to the feeding arteries through the NO-dependent modulation of inter-endothelial gap junctions that are opened in upstream arteriolar vessels and closed in the downstream venular microvessels [50]. Recent evidence suggests that EDH is also supported by the Ca^2+^-activated Cl^−^ channel TMEM16A [40], whereas TRPC3 may provide an additional Ca^2+^ entry pathway recruited by G_q_PCRs to sustain SK_Ca_/IK_Ca_ activation [51] (Figure 2 and Figure 3).

**Figure 4:**
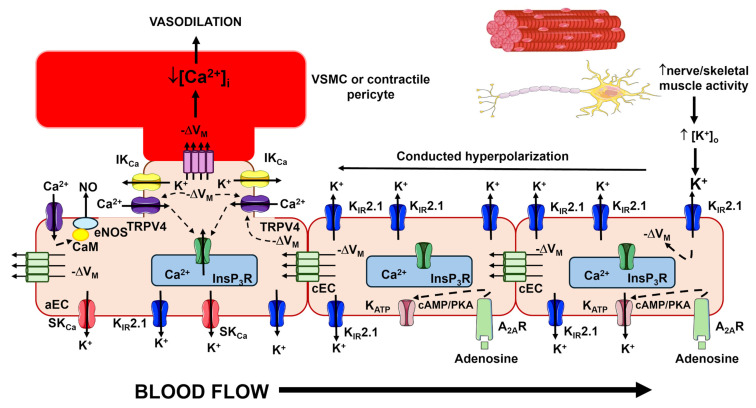
Proposed mechanism of endothelium-dependent hyperpolarization (EDH) in microcirculation. Enhanced neuronal or skeletal muscle activity promotes the accumulation of K^+^ in the extracellular space (up to 8–10 mM). Elevated extracellular K^+^ ([K^+^]_0_) activates K_IR_2.1 channels, inducing membrane hyperpolarization (−ΔV_m_) in capillary endothelial cells (cECs). The hyperpolarizing signal rapidly propagates through homocellular gap-junctions and reaches the arteriole-capillary transition zone, which is ensheathed by contractile pericytes, as well as the endothelium of terminal arterioles (aEC). Herein, EDH increases the electrochemical gradient for Ca^2+^ entry through TRPV4 channels, which triggers Ca^2+^-induced Ca^2+^ release (CICR) via InsP_3_ receptors (InsP_3_Rs) in the ER. The resulting increase in [Ca^2+^]_i_ activates small- and intermediate-conductance Ca^2+^-activated K^+^ channels (SK_Ca_/IK_Ca_). The overall endothelial hyperpolarization, supported by both K_IR_2.1 and SK_Ca_/IK_Ca_ channels, is electrotonically propagated through heterocellular gap junctions to overlying pericytes/VSMCs, leading to vasodilation and redirection of blood flow toward downstream capillaries. Additionally, the enhanced endothelial Ca^2+^ signaling activity mediated by TRPV4 channels and InsP_3_Rs may stimulate eNOS to elicit NO-dependent local vasodilation. Endothelial electrical signals in capillary microcirculation may also be triggered, but not retrogradely propagated, by adenosine, which activates K_ATP_ channels through the G_s_﻿-coupled A_2A_ receptor and cyclic adenosine monophosphate (cAMP)/protein kinase A (PKA) pathway. This schematic is based upon the following references: [1,4,6,9,37,57,68,69,70,71,72,73].

In capillaries, EDH is predominantly sustained by inwardly rectifying K^+^ (K_IR_2.1) and ATP-sensitive K^+^ (K_ATP_) channels, which enable endothelial cells to sense local metabolic activity [4,9,56,57,58]. K_IR_2.1 channels are activated by modest increases in extracellular K^+^, originating either from SK_Ca_/IK_Ca_-mediated K^+^ efflux in upstream arterioles [44,59] or from nearby excitable parenchymal cells in capillaries (Figure 4) [57,58,60,61,62,63]. This activation triggers robust endothelial hyperpolarization that propagates retrogradely to upstream arterioles and is transmitted to the overlying VSMCs/pericytes through MEGJs, thereby promoting vasorelaxation (Figure 4) [4,57,58,60,62,63]. Endothelial KATP channels provide an additional metabolic sensing mechanism; they are gated by decreased ATP/ADP ratio [64,65,66,67] or by adenosine [9,58] via adenosine 2A (A_2A_) receptors/cAMP/protein kinase A (PKA) signaling, thereby contributing to activity-dependent increases in local blood flow (Figure 4) [64,65].

Thus, EDH emerges as a spatially organized signaling pathway wherein SK_Ca_/IK_Ca_ channels dominate arteriolar responses, while K_IR_2.1 and K_ATP_ channels mediate capillary-driven metabolic sensing and retrograde vasodilation. This coordinated mechanism facilitates blood flow redistribution toward metabolically active regions through the relaxation of arteriolar smooth muscle cells and pericytes at the ACT zone (Figure 4).

### 2.3. The Interplay Among Endothelial Ca^2+^ Signals, SK_Ca_/IK_Ca_ Channels and eNOS

The spatiotemporal profile of endothelial Ca^2+^ signals activates different Ca^2+^-sensitive vasorelaxant pathways depending on the source of Ca^2+^ mobilization into the cytosol [3]. MEPs constitute a signaling microdomain essential for regulating vascular tone in resistance arteries, as depicted in Figure 3 [12,13,46,49]. Accordingly, InsP_3_-dependent repetitive Ca^2+^ release events, spatially restricted to MEPs, mediate acetylcholine-induced vasorelaxation in murine small mesenteric arteries by activating IK_Ca_ channels [42]. Furthermore, endothelial InsP_3_Rs proximal to MEPs coordinate myo-endothelial feedback in response to VSMC activation [46]. The bolus of InsP_3_ produced within VSMCs upon α1-adrenergic receptor stimulation is transmitted to adjacent vascular endothelial cells through MEGJs, thereby inducing local InsP_3_-dependent ER Ca^2+^ mobilization and EDH activation [74,75]. The myo-endothelial feedback can also be elicited by VSMC depolarization and Ca^2+^ entry through voltage-gated L-type Ca^2+^ channels; the subsequent Ca^2+^ diffusion through MEGJs triggers InsP_3_-driven CICR and activates EDH [76]. InsP_3_-dependent ER Ca^2+^ release events may propagate to upstream sites over distances of up to ~1 mm, traveling as intercellular Ca^2+^ waves that follow the initial hyperpolarizing signal, thereby enhancing and prolonging vasodilation [77,78,79,80,81]. These inter-endothelial Ca^2+^ waves are sustained by the diffusion of InsP_3_ and/or Ca^2+^ through homocellular gap junctions [7,78,82,83,84,85]. However, recent studies suggest that wave propagation can be driven by an “outside-in signaling” process, wherein plasma membrane InsP_3_Rs in the initiating cell physically interact with and activate G_q_PCRs on the adjacent cell [80,81]. Finally, InsP_3_-dependent ER Ca^2+^ depletion leads to SOCE activation. Preliminary evidence suggests that SOCE is weakly coupled to endothelial SK_Ca_/IK_Ca_ channels, but not to eNOS, in resistance arteries [3,86,87], although the specific role played of SOCE at MEPs remains to be fully elucidated.

In contrast, SOCE supports endothelial Ca^2+^ signals and is physically coupled to eNOS in large arteries [3,19,88,89]. Briefly, eNOS drives endothelium-dependent NO production in response to physiological stimuli, including chemical (e.g., acetylcholine, bradykinin, histamine) and physical (e.g., shear stress) signals [18,90,91,92]. eNOS is concentrated in caveolin 1-enriched caveolae [93,94], which maintain the enzyme in close proximity with Ca^2+^-permeable channels [95,96,97,98,99,100,101,102], such as Orai1, TRP Vanilloid 4 (TRPV4; see Section 2.4) and Piezo1 (see Section 2.5). While caveolin 1 exerts tonic inhibition on eNOS, extracellular Ca^2+^ entry stimulates calmodulin (CaM), which displaces caveolin 1 and triggers NO production. Endothelial-derived NO diffuses to the adjacent VSMCs, activating the soluble guanylate cyclase (sGC)/cyclic GMP (cGMP)/protein kinase G (PKG) signaling pathway to elicit vasorelaxation and reduce MAP [7,19]. Recent reports suggest that sGC may regulate additional vasorelaxant pathways via transnitrosation, affecting both EDH and endothelial Ca^2+^ dynamics in mouse mesenteric resistance vessels [103,104]. Furthermore, evidence from hamster pouch arterioles indicates that the upstream propagation of EDH is sensitive to NO, which exerts a negative feedback on ascending vasodilation [105]. In large arteries, SOCE serves as the primary Ca^2+^ source for agonist-induced NO release [95,96,102,106]; however, evidence for eNOS activation by SOCE in resistance arteries remains elusive [19]. In these vessels, eNOS activation at MEPs is largely mediated by InsP_3_-dependent local Ca^2+^ release events [42]. While NO signaling in resistance arteries contributes to MAP regulation, it appears less prominent than EDH in the control of vascular resistance [3,12,13].

### 2.4. TRP Channels: Focus on TRPV1 and TRPV4

The mammalian TRP superfamily comprises 28 members, which are classified into six sub-families based on gene homology: canonical (TRPC1–7), melastatin (TRPM1–8), vanilloid (TRPV1–6), ankyrin (TRPA1), polycystin (TRPP), and mucolipin (TRPML1–3). Within the TRPP sub-family, which consists of eight members, only TRPP2, TRPP3, and TRPP5 serve as ion channels rather than membrane scaffold proteins [107,108,109]. Most TRP channels conduct Na^+^, K^+^, and Ca^2+^, thereby inducing membrane depolarization and Ca^2+^ influx [107,108,109,110], which can be further amplified by the recruitment of InsP_3_Rs via CICR [111,112,113]. Physiologically, endothelial TRP channels function as polymodal sensors of diverse cellular and environmental cues, including reactive oxygen species (ROS), gasotransmitters (e.g., NO and hydrogen sulphide), arachidonic acid, extracellular Mg^2+^ or pH, temperature, laminar shear stress, osmotic pressure, and pulsatile stretch. Moreover, they are sensitive to dietary agonists, such as capsaicin, menthol, carvacrol, eugenol, and allyl isothiocyanate [20,91,114,115,116,117,118,119,120,121].

The role of endothelial TRP channels in the control of microcirculation, particularly TRPV4 and TRPV1, has been mainly assessed in the mesenteric circulation and brain microvasculature of rodents. Briefly, the polymodal TRPV4 channel is regarded as a critical regulator of Ca^2+^ entry in both the vascular beds [68,69,100,122,123,124]. TRPV4 integrates a variety of chemical and physical cues, including PLCβ-dependent hydrolysis of PIP_2_ (Figure 2), phospholipase A2-mediated generation of AA and EET acids, laminar shear stress, pulsatile stretch and osmotic stress [91,119,123,124,125,126,127,128]. TRPV4-mediated Ca^2+^ entry generates local increases in submembrane Ca^2+^ at MEPs, which activate SK_Ca_/IK_Ca_ channels and induce EDH (Figure 3 and Figure 4) [122]. Consistently, TRPV4-dependent EDH activation mediates acetylcholine-induced vasodilation and blood pressure reduction [48]. Furthermore, the systemic activation of TRPV4 with the synthetic agonist GSK 1016790A causes a marked decrease in MAP in mice [129]. This signaling pathway also supports vasodilation induced by low shear stress in mouse mesenteric arteries [119] and by low intravascular pressure in rat cremaster arteries [126]. Finally, endothelial TRPV4 channels mediate the myo-endothelial feedback triggered by VSMC α1-adrenergic stimulation to limit vasoconstriction [130]. Notably, while TRPV4 is coupled with eNOS in mouse resistance arterioles [100], but NO signaling is severely limited by the hemoglobin alpha (Hbα), which scavenges NO before it elicits vasorelaxation [131,132]. Conversely, in the mouse brain microcirculation, TRPV4-mediated Ca^2+^ entry sustains the intracellular Ca^2+^ signals produced by G_q_PCR-dependent stimulation of InsP_3_Rs at the ACT zone, thereby promoting NO release (Figure 4) [68,124]. This influx can be further enhanced by K_IR_2.1-mediated endothelial hyperpolarization, which rapidly propagates from distal capillary regions and increases the driving-force for Ca^2+^ entry (Figure 4) [69].

TRPV1 provides another example of a polymodal cation channel, activated by temperatures above 41 °C, acidification, intracellular ROS, endocannabinoids (e.g., anandamide), spider-derived vanillotoxins, and various herbal agonists, such as capsaicin, evodiamine and gingerol [117,133,134,135]. TRPV1 activation primarily causes NO release to induce vasodilation [117]. Indeed, TRPV1 stimulation by the capsaicin derivative VOA (N-(4-O-[2-methoxy, phenoxyethylaminobutyl]-3-methoxy benzyl)-nonamide) significantly reduces MAP in both normotensive and spontaneously hypertensive (SHR) rats [136]. Ex vivo and in vitro investigations showed that extracellular Ca^2+^ entry through endothelial TRPV1 channels activates eNOS and stimulates NO release [136]. A subsequent study confirmed that long-term capsaicin administration reduces arterial pressure in SHR rats by stimulating NO release [137]. Similarly, capsaicin induces a NO-dependent decrease in coronary vascular resistance in rats [138] and the genetic deletion of TRPV1 reduces capsaicin-induced increase in myocardial blood flow in mice [139]. Finally, capsaicin stimulates endothelial TRPV1 channels to induce vasorelaxation in mouse mesenteric arteries [140] and to boost acetylcholine-induced vasorelaxation in mouse skeletal muscle feed arteries [141]. The subcellular localization of TRPV1 remains incompletely defined, suggesting it may recruit Ca^2+^-dependent vasorelaxant pathways outside the MEPs.

In summary, these findings indicate that TRPV4 and TRPV1 contribute to microvascular regulation through distinct but complementary endothelial signaling pathways: TRPV4 preferentially couples Ca^2+^ entry to EDH-dependent mechanisms, whereas TRPV1 predominantly engages NO-mediated vasorelaxation. The sensitivity of both channels to endogenous, dietary or synthetic agonists supports their potential as pharmacological targets for modulating microvascular blood flow under pathological conditions (see Section 4).

### 2.5. Piezo1 Channels

The mechanosensitive Piezo channels act as *bona fide* mechanosensors in mammalian cells: Piezo1 is the primary mechanosensitive channel in the cardiovascular system, whereas Piezo2 functions as a touch receptor and proprioceptor in the peripheral nervous system [118,142,143]. Piezo1 channels are non-selective cation channels that mediate endothelial depolarization and Ca^2+^ entry in response to membrane stretch and blood flow-induced shear stress [118,143]. Under low shear stress conditions, Piezo1-mediated Ca^2+^ entry induces NO release, thereby resulting in the vasodilation of mouse mesenteric arteries and reducing MAP [90,144]. Additionally, the pharmacological activation of Piezo1 channels with the shear stress mimetic Yoda1 stimulates SK_Ca_/IK_Ca_ channels to promote EDH in the mouse microvascular mesenteric bed [145]. Intriguingly, under increased laminar shear stress during physical exercise, endothelial Piezo1-mediated depolarization outweighs the recruitment of the Ca^2+^-dependent vasorelaxant pathways. This electrical signal is transmitted to the adjacent VSMCs in mouse mesenteric resistance arterioles, triggering contraction and reducing blood flow to downstream capillaries [146]. According to this model, endothelial depolarization spreads to VSMCs though MEGJs and induces contraction by activating voltage-gated L-type Ca^2+^ channels [146]. This effect is vascular bed-specific, as Piezo1 activation does not interfere with the vasorelaxing mechanisms in the saphenous or carotid artery [146]. Consequently, it has been proposed that Piezo1 activation constricts mesenteric resistance arteries to redirect blood flow away from the gastrointestinal tract to other organs, such as skeletal muscle [146].

## 3. The Role of Endothelial Ion Channels in the Control of Human Microcirculation

Evaluating the role of endothelial ion channels in the in vivo regulation of human microcirculation poses a considerable experimental challenge. Nevertheless, non-invasive approaches, such as Laser-Doppler Flowmetry (LDF) and Laser Speckle Contrast Imaging (LSCI), have been designed to assess human microvascular reactivity [147,148]. In this section, we summarize current evidence on endothelial ion channel function derived from direct measurements of microvascular reactivity in human subjects. When appropriate, this information is integrated with data obtained from direct videomicroscopy of cannulated, pressurized arterioles and cultured endothelial cells. The skin and forearm muscle microcirculations are the vascular beds most easily accessible to LDF and LSCI technologies and are also suitable for topical, intradermal, or intramuscular delivery of drugs targeting specific ion channels [149].

### 3.1. Cutaneous Microcirculation

The cutaneous microcirculation exhibits high vascular density and plays a critical role in maintaining core body temperature through its thermoregulatory function. In addition, the skin microvasculature is responsible for the excretion of minerals, water, and drugs, while providing protection against external agents, such as extreme temperature fluctuations, ultraviolet irradiation, and microorganisms [150]. The cutaneous microcirculation is structured into two main plexuses, specifically the subdermal and dermal plexus; these run parallel to the skin surface and allow for the assessment of endothelial function through LDF [151]. These plexuses are connected by ascending arterioles, which branch to form microvascular networks around hair follicles and sweat glands, and descending venules [150]. The subdermal plexus contains an extensive network of terminal arterioles, capillaries and venules, with capillaries organized into papillary loops that provide a large surface area for heat exchange [152]. Arteriovenous anastomoses (AVAs) are located in the dermis of glabrous skin, bypassing the high-resistance arterioles and capillaries of the papillary loops to drain into the veins of the subdermal plexus. AVAs function to rapidly increase dermal blood flow, thereby favoring the dissipation of excessive heat and reducing body temperature [150,152].

Owing to its high accessibility, the cutaneous microcirculation is regarded as a valuable experimental model to investigate systemic microvascular function [151,153]. Several reactivity tests have been designed to investigate blood flow regulation in this district, including post-occlusion reactive hyperemia (PORH), pressure-induced vasodilation, thermal challenges and the local infusion of pharmacological agents [151]. Current knowledge suggests that endothelial ion channels play a crucial role in the hemodynamic response to elevated body temperature, local heating of human skin, and PORH (Table 1 at the end of Section 3.1).

#### 3.1.1. Whole Body Heat Stress

The regulation of skin blood flow (SkBF) is under tight sympathetic and afferent sensory control, while also being modulated by endothelial, myogenic, and metabolic factors [150]. In humans, the autonomic sympathetic nervous system innervates arterioles and AVAs of the nonglabrous skin through both cholinergic and adrenergic fibers. The adrenergic vasoconstrictor system maintains the basal vasoconstrictor tone exerted on the cutaneous microcirculation during thermoneutral conditions and is further enhanced by cold exposure. Body temperature increases when individuals are exposed to high-temperature environments or when heat production rises during shivering or physical exercise [154]. This thermal challenge triggers sweating and cutaneous vasodilation, causing SkBF to rapidly increase from ~250 mL/min to as much as ~6–8 L/min, thereby accelerating heat dissipation [155]. The initial response to elevated body temperature is mediated by the withdrawal of sympathetic adrenergic vasoconstrictor tone, followed by the reflex activation of the cutaneous active vasodilation (CAVD) system. This system is driven by the sympathetic cholinergic vasodilatory pathway and is largely responsible for the subsequent increase in SkBF. In contrast, glabrous skin lacks a functional cholinergic component, and vasodilation in response to heat is exclusively mediated by the reduction in adrenergic vasoconstrictor activity [150,152,154,156].

#### 3.1.2. The Role of Endothelial-Derived NO in CAVD: Acetylcholine and Histamine

**Figure 5:**
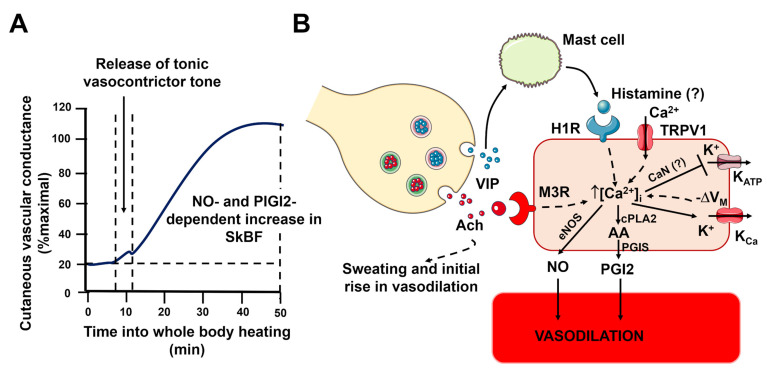
Summary of the main endothelial signaling mechanisms contributing to cutaneous active vasodilation. (**A**), a representative tracing of the skin blood flow (SkBF) response to whole-body heating is shown, illustrating baseline SkBF (expressed as a percentage of maximal cutaneous vascular conductance), the initial increase reflecting the release of tonic vasoconstrictor tone, and the subsequent rise in SkBF attributed to active vasodilation mediated by sympathetic cholinergic nerves. (**B**), a schematic overview of the current model of cutaneous active vasodilation is presented. Acetylcholine (ACh) is released and contributes to the early phase of active vasodilation by activating endothelial muscarinic type 3 receptors (M3Rs), while simultaneously stimulating sweat gland activity. Vasoactive intestinal peptide (VIP) is among the putative cotransmitters released with Ach and is thought to stimulate mast cells to release histamine. Histamine binds to and activates endothelial histamine type 1 receptors (H1Rs). Evidence also supports the role of Transient Receptor Potential Vanilloid 1 (TRPV1). These signaling pathways are predicted to elicit an increase in intracellular Ca^2+^ concentration ([Ca^2+^]_i_ in dermal microvascular endothelial cells, leading to Ca^2+^-dependent production of nitric oxide (NO) and prostacyclin (prostaglandin I2), which, respectively, account for ~40–50% and ~26% of peak vasodilation [154]. Emerging evidence suggests that dermal microvascular endothelial cells also express small- and intermediate-conductance Ca^2+^-activated K^+^ channels (K_Ca_) channels, which respond to increases in [Ca^2+^]_i_ and enhance the electrochemical gradient for Ca^2+^ entry through plasma membrane Ca^2+^-permeable channels (e.g., TRPV1). This additional Ca^2+^ influx could stimulate calcineurin (CaN), which in turn inhibits ATP-dependent K^+^ channels (K_ATP_). K_ATP_ channels are thought to serve as a backup vasorelaxant pathway that emerges when K_Ca_ channels are inhibited (e.g., by tetraethylammonium).

According to the most widely accepted model, CAVD is initiated by endothelium-derived NO production in response to acetylcholine released from the sympathetic cholinergic terminals. Acetylcholine accounts for ~20% of cutaneous vasodilation during whole body heating, while the remaining 80% is attributed to a vasorelaxant mediator that is co-released with acetylcholine but has yet to be clearly identified (Figure 5) [150,152,154,156,157]. It has been estimated that NO accounts for ~40–50% of peak vasodilation, while prostacyclin contributes up to ~26% of active vasodilation [154].

The cholinergic component of the NO-mediated vasodilatory response is inhibited by atropine, supporting the involvement of muscarinic receptors [157,158,159,160,161,162,163,164]. M1, M3 and M5 muscarinic receptors are G_q_PCRs that are able to elicit an increase in endothelial [Ca^2+^]_i_ by triggering PLCβ signaling as described in Section 2.2 [83,92,165,166,167]. Although the specific muscarinic receptor isoforms expressed in human cutaneous endothelial cells remain incompletely defined, preliminary evidence reveals that M3 receptors are expressed in endothelial cells derived from human induced pluripotent stem cells (hiPSCs) that were in turn generated from human skin fibroblasts [168]. Acetylcholine can also activate the endothelial nicotinic acetylcholine receptors (nAchRs) [169,170], which are expressed in human cutaneous endothelial cells [171,172]. The vascular endothelium expresses neuronal nAChRs that consist of either α7-α9 homopentamers or heteropentamers comprising at least one α (α2–α6) and one β (β2–β4) subunit [169,173]. The molecular profile of cutaneous nAchRs is compatible with the expression of α7-nAchRs [172], which display high Ca^2+^ permeability [173]. Notably, several investigations suggest that endothelial nAchRs are not involved in CAVD, but rather regulate the cutaneous vascular tone under normothermic conditions by reducing NO bioavailability through an as-yet-unidentified mechanism [174,175,176]. Interestingly, aging selectively reduces nicotine-induced cutaneous vasodilation in women but not in men [177].

The cholinergic co-transmission theory has prompted the search for the additional neurotransmitters mediating active vasodilation during CAVD. According to the most popular model [150,152,156], vasoactive intestinal peptide (VIP) is released from sympathetic cholinergic nerves, thereby stimulating mast cells to release histamine (Figure 5). Histamine, in turn, binds to and activates H1R, a G_q_PCR that stimulates NO release and cutaneous vasodilation (Figure 5) [178,179]. H1R is known to induce Ca^2+^-dependent eNOS activation in human vascular endothelial cells [180,181,182]. A recent investigation unveiled that histamine induces endothelial Ca^2+^ oscillations by promoting the rhythmic mobilization of the ER and lysosomal Ca^2+^ stores via InsP_3_R3 and TPCs, respectively. The subsequent depletion of the ER Ca^2+^ pool leads to SOCE activation, which maintains histamine-induced intracellular Ca^2+^ oscillations over time [18,183]. Future studies using isolated cutaneous microvascular endothelial cells are required to confirm the role of InsP_3_Rs, TPCs and SOCE in the Ca^2+^-dependent recruitment of eNOS by acetylcholine and histamine. Neurokinin-1 (NK1) receptors also support CAVD in the human skin microcirculation [184]. NK1 receptors are GPCRs that can increase the endothelial [Ca^2+^]_i_ through a mechanism that involves InsP_3_-dependent ER Ca^2+^ release and, potentially, SOCE [185,186,187]. An early investigation demonstrated that the desensitization of NK1 receptors through repeated applications of substance P reduced the cutaneous active vasodilator response to heat stress. Although Substance P can activate eNOS [186,188], the modulation of CAVD also involves an NO-independent component [184]. Intriguingly, substance P can trigger EDH by activating SK_Ca_ channels in porcine coronary arteries [189,190,191]. However, as discussed below, EDH is unlikely to contribute significantly to CAVD in human skin microcirculation. In addition, it is unclear whether substance P, which can be released by endothelial cells undergoing heat stress [156], or other tachykinins, such as neurokinin A and neurokinin B, are the true physiological agonists of cutaneous NK1 receptors [154].

#### 3.1.3. The Role of EDH in CAVD

The role of endothelial K^+^ channels in CAVD has been primarily investigated by assessing the inhibitory effect of high concentrations of tetraethylammonium (TEA), a broad-spectrum K^+^ channel blocker [192,193,194,195]. TEA inhibits several types of K^+^ channels, including as big-conductance Ca^2+^-activated K^+^ (BK_Ca_) channels [195,196], voltage-gated K^+^ channels [193,197] and SK_Ca_/IK_Ca_ channels [195]. Although its lack of specificity has long been recognized [150], TEA is still regarded in the SkBF field as a non-selective blocker of Ca^2+^-activated K^+^ channels. Notably, TEA does not affect cutaneous vasodilation induced by passive heating. Future studies should therefore assess whether CAVD is affected by more specific inhibitors of SK_Ca_ channels, such as UCL 1684 and AP14145, or IK_Ca_ channels, such as TRAM-34 and Senicapoc [198,199]. Unexpectedly, when TEA was administered in combination with the eNOS inhibitor L-NAME, it rescued the reduction in the active vasodilatory response caused by eNOS inhibition [200]. This observation suggests that the combined inhibition of K_Ca_ channels and NO production unmasks the activation of alternative vasorelaxant mechanisms. Consistent with this hypothesis, the infusion of barium chloride (BaCl_2_) restored CAVD to the levels measured in the presence of L-NAME alone [200]. BaCl_2_ inhibits both K_IR_ and K_ATP_ channels [9,57,59,201]. We, therefore, propose that TEA acts by preventing endothelial hyperpolarization during CAVD, thereby reducing the driving force for Ca^2+^ entry during acetylcholine- and/or histamine-induced Ca^2+^ signaling (Figure 5) [43,202]. Elevated [Ca^2+^]_i_ can recruit the Ca^2+^-dependent phosphatase calcineurin, which inhibits K_ATP_ channels [203,204], whereas Ca^2+^-dependent modulation of K_IR_ channels has never been reported. Therefore, we suggest that K_ATP_ channels may serve as a backup vasorelaxant pathway that replaces NO when SK_Ca_/IK_Ca_ channels are impaired and excessive Ca^2+^ entry into cutaneous microvascular endothelial cells is prevented (Figure 5). This hypothesis requires direct experimental validation. First, it remains to be confirmed whether TEA acts specifically by inhibiting SK_Ca_/IK_Ca_ channels. Second, although SK_Ca_/IK_Ca_ currents have been recorded in human dermal endothelial cells [205], there is currently no evidence supporting the expression of K_IR_2.1 and K_ATP_ channels in this cell type. Future investigations should therefore determine whether more selective K_ATP_ channel antagonists, such as tolbutamide, glibenclamide, or glinides, affect the active cutaneous vasodilator response to whole body heating [206].

#### 3.1.4. The Role of Endothelial TRP Channels in CAVD: Evidence for TRPV1 Involvement

Several members of the TRP channel superfamily serve as thermosensors across a broad range of physiological and pathological temperatures [107,108,109]. SkBF increases in response to the pharmacological activation of multiple TRP isoforms that are expressed in human skin endothelial cells, such as TRPV1 by capsaicin [207,208,209], TRPV4 by GSK1016790 A [210], and TRPA1 by cinnamaldehyde [211,212]. TRPV1 is activated by temperatures exceeding 41 °C and can induce NO-dependent vasorelaxation [116,117]. It has long been known that TRPV1 is present in cutaneous sensory afferents, thereby controlling the release of vasorelaxing mediators, such as calcitonin gene-related peptide (CGRP) and NO [150,152]. Nevertheless, earlier studies suggested that the reflex cutaneous vasodilator response to hyperthermia may also be mediated by endothelial TRPV1 channels [213]. A subsequent investigation exploiting the non-selective TRPV1 antagonist capsazepine demonstrated that TRPV1-dependent NO release contributes to reflex cutaneous vasodilation during whole-body heat stress (Figure 5) [214]. In vitro studies further showed that TRPV1 is expressed [215] and functional [216] in human dermal microvascular endothelial cells. Future studies should therefore assess the impact of more specific TRPV1 inhibitors, such as ABT-102, AZD1386, JNJ-39439335 and SB-366791 [112,217], on CAVD. TRPV3 channels detect moderate warmth, with a temperature threshold between 32 °C and 39 °C [218], and are capable of triggering EDH [219]. Within the vascular system, endothelial TRPV3 channels have been detected exclusively in the brain microcirculation [114,219], whereas they are highly enriched in cutaneous sensory nerve endings and keratinocytes [220]. Accordingly, current evidence argues against a role for dermal endothelial cells in the TRPV3-mediated CAVD response to heat exposure [150,221,222]. TRPV4 channels can also function as thermosensitive channels, being activated by temperatures ranging between 27 °C and 34 °C [223,224]. TRPV4 is abundantly expressed in human dermal microvascular endothelial cells [225,226], where TRPV4-mediated Ca^2+^ entry elicits only modest NO production [227]. However, recent evidence indicates that TRPV4 channels do not contribute to the increase in SkBF induced by whole-body passive heating [228].

Human TRPA1 channels display an intrinsic U-shaped thermosensitivity, as they are activated by both cold (~10 °C) and hot (~30 °C) temperatures [229]. Hattori and coworkers reported that TRPA1 contributes to cutaneous vasodilation during exercise in the heat, but only under conditions of NO signaling inhibition [230]. The cellular localization of TRPA1 was not addressed in that study. In addition to human dermal microvascular endothelial cells, TRPA1 channels are also expressed in cutaneous sensory nerve endings [231] and keratinocytes [232]. Accordingly, future studies are required to verify whether endothelial TRPA1 channels contribute to CAVD. At present, evidence supports only endothelial TRPV1 involvement in CAVD.

#### 3.1.5. The Role of Endothelial Ion Channels in Vasodilation to Local Heating

An increase in SkBF serves as a protective mechanism against excessive local heating of the skin [150]. In non-glabrous skin, a local increase in temperature causes a rapid initial peak in SkBF, which is followed by a brief decline, known as the nadir, and by a secondary rise to a sustained plateau phase [233]. Current evidence suggests that endothelial ion channels are critically involved in the development and maintenance of this plateau phase. A neuronal reflex likely drives the early peak increase in SkBF upon the activation of TRPV1 channels located on afferent sensory nerves [233,234]. The subsequent plateau hyperemia is supported by endothelial TRPV1 channels, which promote NO release [233,234], as well as by EDH, the precise molecular identity of which remains incompletely defined [235]. Notably, half of the EDH-dependent component of thermal hyperemia is sensitive to the pharmacological blockade of EET production with sulfaphenazole [235]. Future studies should therefore determine whether EETs promote vasodilation by stimulating TRPV4-mediated Ca^2+^ entry and SK_Ca_/IK_Ca_ channel activation, as demonstrated in several animal models [236,237,238]. Interestingly, the administration of theophylline, a non-selective antagonist of A_1_ and A_2_ receptors, further reduces cutaneous vasodilation induced by local heating of human skin, suggesting that adenosine-induced active vasodilation augments both NO- and EDH-dependent responses [239]. Adenosine may exert these effects by activating endothelial and/or VSMC K_ATP_ channels [64,65]. However, the relative contribution of these pathways remains to be clarified.

#### 3.1.6. The Putative Role of Endothelial Ion Channels in Post-Occlusion Reactive Hyperemia (PORH)

The rapid increase in SkBF observed following a brief (3–5 min) arterial occlusion is referred to as PORH [150]. A local axon reflex represents the primary mediator of PORH in the human forearm [150,240,241,242]. Neither NO nor cyclooxygenase appear to contribute to PORH [159,243]; however, this reactive hyperemia is markedly reduced by the administration of TEA and glibenclamide [243]. These findings suggest that Ca^2+^-activated K^+^ channels and K_ATP_ channels, located in either vascular endothelial cells or VSMCs, contribute to PORH. The increase in blood flow occurring after the removal of arterial occlusion may activate mechanosensitive endothelial cation channels [118,150,244], such as Piezo1 [90,245] and TRPV4 [119,246,247] channels, both of which are capable of recruiting SK_Ca_/IK_Ca_ channels [145,236,237,238]. The specific role of Piezo1 and TRPV4 channels in PORH remains to be directly investigated. Notably, Piezo1 channels have recently been identified in human cutaneous blood vessels [248], and PORH is also attenuated by the pharmacological inhibition of EET production [249]. In line with this observation, studies in mouse carotid arteries demonstrate that flow-induced vasodilation requires EET-dependent TRPV4 activation [250]. Future investigations should therefore determine whether the EET/TRPV4/EDH signaling pathway also contributes to PORH.

**Table 1:** The role of endothelial ion channels in the regulation of human skin microcirculation.

Ion Channel	Physiological Mechanism	Vasorelaxing Signaling Pathway	Role	References
SK_Ca_/IK_Ca_ channels	CAVD	Controls the release of a NO-independent mechanism	Likely	[200]
SK_Ca_/IK_Ca_ channels	Local heating	EDH	Yes	[235]
SK_Ca_/IK_Ca_ channels	PORH	EDH	Likely	[243]
K_IR_2.1 channels	CAVD	EDH	Unknown	
K_ATP_ channels	CAVD	EDH	Unknown	
K_ATP_ channels	Local heating	EDH	No evident role	
K_ATP_ channels	PORH	EDH	Yes	[243]
TRPV1	CAVD	NO release		[214]
TRPV1	Local heating	NO release EDH		[233,234] [235]
TRPV3	CAVD		No evident role	[150,221,222]
TRPV4	CAVD		No role	[228]
TRPV4	Local heating		No evident role	
TRPA1	CAVD	Unclear endothelial localization	Yes	[230]

Abbreviations: CAVD: cutaneous active vasodilation; EDH: endothelium-dependent hyperpolarization; NO: nitric oxide; PORH: post-occlusive reactive hyperemia.

### 3.2. Skeletal Muscle Blood Flow

During physical exercise, skeletal muscle blood flow increases dramatically to meet the elevated metabolic demands of contracting muscle fibers. This process is initiated by rapid vasodilation occurring almost immediately at the onset of muscle contraction, primarily mediated by a decrease in vascular resistance within feed arteries and arterioles [251,252]. The hemodynamic response to physical exercise consists of a rapid and transient (∼1 min) increase in blood flow, followed by a transition phase and ultimately by a rise to a sustained plateau, during which blood flow is closely matched to metabolic requirements [253]. Regardless of whether muscle fibers contract proximal to an arteriole or a capillary, the initial vasodilatory response must propagate upstream to induce dilation of the terminal arteriole, thereby supporting perfusion of the capillary unit that supplies each activated motor unit [251,252]. Blood flow to skeletal muscles can increase by more than 100-fold during the transition from inactivity to maximal aerobic exercise due to functional sympatholysis. This mechanism counteracts sympathetic α1-adrenergic vasoconstriction in actively contracting skeletal muscles to meet increased metabolic demands [254]. Growing evidence indicates that the ion signaling machinery in endothelial cells and VSMCs plays a critical role in functional sympatholysis through EDH-dependent mechanisms. In contrast, the extent to which other endothelial vasodilatory mediators, such as NO and prostaglandins, contribute to exercise hyperemia remains incompletely defined [255,256,257,258]. The endothelial ion channels that may regulate functional sympatholysis and exercise hyperemia in the human skeletal muscle microcirculation are summarized in Table 2 at the end of Section 3.2.

#### 3.2.1. Endothelium-Derived NO Is Unlikely to Be the Primary Mediator of Functional Sympatholysis but Supports the Matched Phase of Exercise Hyperemia

Immediate vasodilation following a single muscle contraction in humans is unlikely to be primarily mediated by NO. An elegant early model proposed that acetylcholine “spillover” from motor nerve terminals near neuromuscular junctions could stimulate eNOS, leading to NO-dependent vasodilation through the activation of endothelial M3 receptors [251,257]. Subsequent studies, however, showed that acetylcholine spillover from the neuromuscular junction is not required to trigger contraction-induced vasodilation [255,257,259,260]. Likewise, NO has been found to be neither sufficient nor obligatory for functional sympatholysis during physical activity in humans [256,261,262,263]. Nevertheless, endothelium-derived NO contributes to the steady-state (matched) phase of blood flow increase [264,265], which is proportional to the metabolic demand of the contracting muscle [253,256]. NO is unlikely to promote vasorelaxation alone but rather acts in concert with prostaglandins [256]. Consistent with this notion, the simultaneous inhibition of these vasodilatory pathways accounts for ~15–30% of the reduction in the hyperemic response to physical exercise [256].

**Figure 6:**
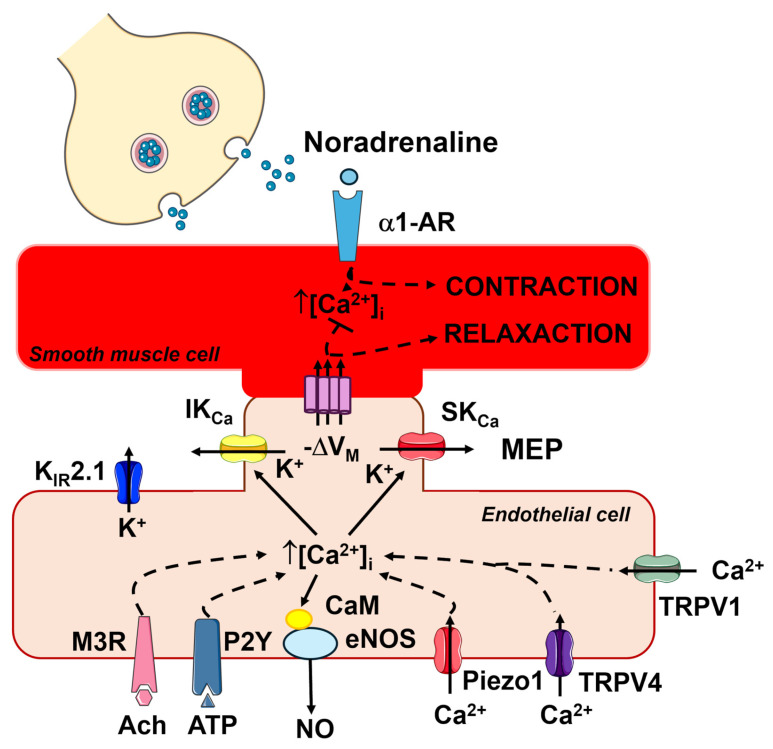
Summary of the main endothelial signaling mechanisms contributing to functional sympatholysis and exercise hyperemia. At the onset of muscle exercise, an increase in sympathetic tone stimulates vascular smooth muscle cell (VSMC) contraction following the activation of the G_q_ protein-coupled α1-adrenergic receptors (α1-ARs). At the same time, an increase in intracellular Ca^2+^ concentration ([Ca^2+^]_i_) can be elicited in vascular endothelial cells by acetylcholine (Ach) and ATP, which, respectively, activate the G_q_ protein-coupled endothelial muscarinic type 3 receptors (M3Rs) and purinergic P2Y receptors. The endothelial Ca^2+^ signal can, in turn, promote endothelium-dependent hyperpolarization (EDH) by stimulating the small- and intermediate-conductance Ca^2+^-activated (SK_Ca_/IK_Ca_) channels. The subsequent efflux of K^+^ activates the inwardly rectifying K_IR_2.1 channel, that amplifies the initial hyperpolarizing signal. The ensuing EDH is then electrotonically transmitted to overlying VSMCs, where it inhibits voltage-gated L-type Ca^2+^ channels and promotes relaxation. The increase in endothelial [Ca^2+^]_i_ also recruits calmodulin (CaM) to stimulate NO release via eNOS activation. NO production can be further supported by Ca^2+^ entry through the mechano-sensitive Piezo1 and TRPV4 channels, which are sensitive to the increase in luminal shear stress that follows the onset of vasodilation. Finally, the local increase in temperature associated with skeletal muscle contraction could activate Ca^2+^ entry through TRPV1 and TRPV4 channels.

NO release can be driven by Ca^2+^ entry through mechanosensitive endothelial channels activated by the rise in luminal shear stress following the onset of vasodilation, including Piezo1 [90,266] and TRPV4 [91,247] channels (Figure 6). As discussed in Section 2.5, studies conducted in different mouse microvascular beds suggest (though do not directly demonstrate) that Piezo1 activation contributes to the increase in blood flow to skeletal muscle during physical activity [146]. Similarly, it has been proposed that regular aerobic activity induces exercise hyperemia via shear stress-mediated TRPV4 activation [267]. Future studies are required to determine whether endothelial Piezo1 and TRPV4 channels operate as partially redundant mechanosensitive Ca^2+^ entry pathways that transduce mechanical stimuli into an effective hemodynamic response to physical exercise. Additionally, NO release in the skeletal muscle microcirculation may be elicited by the direct activation of specific TRP channels in response to local hypoxia and heat production. Emerging evidence suggests that red blood cells (RBCs) function as oxygen sensors and release ATP in response to local hypoxia [268,269]. ATP could, in turn, bind to purinergic P2Y receptors and thereby recruit TRPV4 channels in human skeletal muscle endothelial cells [270,271]. Concurrently, thermosensitive endothelial TRP channels that are strongly coupled with eNOS, such as TRPV1 [117,272] and TRPV4 [127,128], may support heat-induced NO release during exercise hyperemia (Figure 6) [141,269,273]. Notably, both TRPV1 and TRPV4 are also sensitive to multiple mediators released during physical exercise [112,117,274,275], including ROS, protons, and anandamide, making them well suited to couple increased metabolic activity with local blood flow regulation. Intriguingly, TRPV4-mediated NO release was also found to blunt α1-adrenergic receptor-dependent vasoconstriction, suggesting its involvement in functional sympatholysis [141,273], as further discussed below. However, it should be emphasized that direct molecular or cellular evidence for the expression of Piezo1, TRPV1, and/or TRPV4 channels in human skeletal muscle endothelial cells is still lacking.

#### 3.2.2. The Role of EDH and K_IR_ Channels in Functional Sympatholysis and Exercise Hyperemia

Emerging evidence suggests that EDH mediated by the activation of K_IR_ channels is crucial for the full development of the hemodynamic response to physical exercise (Figure 6) [276]. An early investigation showed that blocking endothelial K_IR_ channels with BaCl_2_ reduced the vasodilatory response to a single muscle contraction by ~30–50% [277]. Follow-up reports confirmed that the intra-arterial infusion of BaCl_2_ reduced both the rapid-onset hemodynamic response to handgrip exercise by ~50% and the steady-state response by ~30% [278,279]. Moreover, the activation of K_IR_ channels was shown to couple local vasodilation with the progressive recruitment of skeletal muscle fibers at the onset of exercise and during transitions to higher exercise intensities [280]. However, it remains unclear whether endothelial K_IR_ channels are activated by K^+^ released during skeletal muscle contraction or whether they instead function as signal amplifiers of the canonical EDH pathway [59,276]. Notably, the contribution of upstream SK_Ca_/IK_Ca_ channels within the EDH signaling pathway has yet to be directly investigated in this context [276]. Recently, Hearon and coworkers adopted an alternative pharmacological approach to assess the role of EDH in exercise hyperemia. They showed that the intra-arterial infusion of acetylcholine enhanced the hemodynamic response to handgrip exercise by selectively recruiting K_IR_ channels [281]. This evidence suggests that endothelial SK_Ca_/IK_Ca_ channels may be recruited by acetylcholine via an M3 receptor-mediated increase in [Ca^2+^]_i_, thereby supporting physical exercise-induced vasodilation (Figure 6) [282,283]. An additional autacoid that can promote EDH in the skeletal muscle microcirculation is ATP [268,276]. Intra-arterial ATP administration was found to enhance exercise-induced vasodilation by ~40–50% through a mechanism involving K_IR_2.1 channel activation [284,285]. ATP may recruit K_IR_ channels by activating endothelial P2Y receptors, leading to a biphasic increase in [Ca^2+^]_i_ [282,286] and engaging SK_Ca_/IK_Ca_ channels [55,287]. Preliminary evidence suggests that EDH is impaired and contributes to microvascular dysfunction following cardiopulmonary bypass [288].

Interestingly, intravascular ATP also blunted α1-adrenergic vasoconstriction at the onset of handgrip exercise. Surprisingly, this inhibitory effect of ATP on functional sympatholysis was not affected by the combined inhibition of K_IR_ channels, Na^+^/K^+^ ATPase, NO and prostaglandin production [284]. A series of follow-up reports demonstrated that the infusion of ATP or acetylcholine to stimulate endothelial signaling mitigated α_1_-mediated vasoconstriction during mild-to-moderate handgrip exercise, independently of NO and prostaglandins, in both young [289] and old [290] subjects (Figure 6). These observations led to the proposal that EDH mediated by SK_Ca_/IK_Ca_ channels counteracts functional sympatholysis in contracting muscle and may be further amplified by the recruitment of endothelial K_IR_ channels [276,281,289]. An alternative, though not mutually exclusive, explanation for these findings invokes myo-endothelial feedback mechanisms, as described in Section 2.2 [13,46,49,276,291,292]. An increase in sympathetic tone leads to an increase in VSMC [Ca^2+^]_i_ through α1-adrenergic receptor-mediated stimulation of PLCβ activity and InsP_3_ production. The resulting transfer of InsP_3_ and/or Ca^2+^ to adjacent endothelial cells via MEGJs can, in turn, promote vasodilation by recruiting SK_Ca_/IK_Ca_ channels [291,292] or eNOS [292,293]. Similarly, VSMC depolarization can indirectly recruit the endothelial Ca^2+^-dependent vasorelaxing pathways by activating voltage-dependent Ca^2+^ entry through L-type Ca^2+^ channels, followed by Ca^2+^ diffusion through MEGJs and stimulation of CICR via endothelial InsP_3_Rs [3,76]. Collectively, these feedback mechanisms may attenuate α1-adrenergic receptor-mediated vasoconstriction and contribute to functional sympatholysis.

**Table 2:** The role of endothelial ion channels in the regulation of human skeletal muscle microcirculation.

Ion Channel	Physiological Mechanism	Vasorelaxing Signaling Pathway	Role	References
SK_Ca_/IK_Ca_ channels	Functional sympatholysis	EDH	Likely	[284,289,290]
SK_Ca_/IK_Ca_ channels	Exercise hyperemia	EDH	Likely	[281]
K_IR_2.1 channels	Exercise hyperemia	EDH	Yes	[277,278,279,280]
TRPV1	Exercise hyperemia	NO release	Likely	
TRPV4	Exercise hyperemia	NO release	Likely	[267]
Piezo1	Exercise hyperemia	NO release	Likely	

Abbreviations: EDH: endothelial-dependent hyperpolarization; NO: nitric oxide.

### 3.3. Cerebral Blood Flow (CBF)

**Figure 7:**
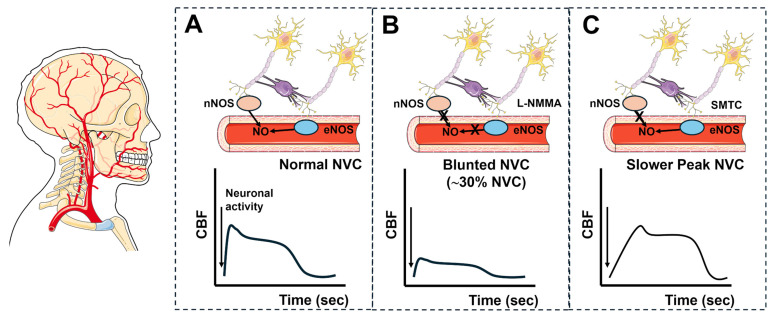
Schematic illustration of the vasorelaxing role of nitric oxide (NO) in human brain microcirculation. (**A**), the increase in cerebral blood flow (CBF) in response to neuronal activity is driven by NO produced by both neuronal NO synthase (nNOS) and endothelial NO synthase (eNOS). (**B**), the non-selective NOS blocker, L-NMMA (*N*^G^-monomethyl-L-arginine), reduces the peak NVC response by ~30%. (**C**), the inhibition of nNOS with SMTC (S-methyl-L-thiocitrulline) reduces the rate of increase in CBF without affecting the overall NVC response. This schematic is based on the following references: [297,298,299,300].

The brain consumes a large amount of energy to support neuronal firing (accounting for ~20% of total body energy), but it is unable to store sufficient fuel to support high-frequency and/or long-lasting ionic signaling. Therefore, the brain microvasculature receives a large fraction of the cardiac output (~15–20%), thereby providing adequate oxygen and nutrient delivery to meet metabolic demands and sustain neuronal signaling and information processing [11,294]. The mechanism by which an increase in neuronal activity is translated into a local elevation in CBF is known as neurovascular coupling (NVC), also referred to as functional hyperemia [11,294]. Current evidence suggests that NO plays a crucial role in NVC in the human brain microcirculation, contributing to both basal cerebrovascular tone [295] and the activity-dependent increase in CBF during cortical stimulation [296,297]. A preliminary investigation reported that visual stimulation elicited a biphasic NVC response in the posterior cerebral artery, consisting of a rapid initial rise in CBF followed by a steady-state elevation in the hemodynamic signal (Figure 7A) [298]. The systemic intravenous infusion of L-NMMA (*N*^G^-monomethyl-L-arginine), a non-selective inhibitor of NOS activity, reduced the peak NVC response by ~30% (Figure 7B) [298]. Selective blockade of neuronal NOS (nNOS) with SMTC (S-methyl-L-thiocitrulline) reduced the rate of the initial rise in CBF in the right hippocampus, parahippocampal gyrus and medial temporal lobe, while exerting a more limited effect on the peak NVC response (Figure 7C) [299]. In addition, systemic infusion of SMTC reduced regional CBF in the right hippocampus and parahippocampal gyrus, accompanied by a decrease in functional connectivity to the left superior parietal lobule [300]. These findings indicate that nNOS is crucial for the regulation of basal CBF and the early phase of the hemodynamic response to cortical stimulation in the human brain microcirculation. Studies in mouse models demonstrate that glutamate released during neuronal activity activates neuronal N-methyl-D-aspartate (NMDA) receptors, leading to Ca^2+^ entry and subsequent nNOS activation [301,302]. The evidence that nNOS inhibition with SMTC only affected the early phase of the hemodynamic response, while the non-selective inhibition of all NOS isoforms with L-NMMA markedly reduced the global increase in CBF, supports the notion that eNOS also contributes to NVC in the human brain microcirculation [303]. A selective eNOS blocker, namely L-NIO (L-N^5^-(1-Iminoethyl) ornithine) [304,305], is also available; however, its effects on NVC in humans have yet to be investigated. Nevertheless, it has been reported that whisker stimulation increases local CBF in the somatosensory cortex by activating endothelial NMDA receptors, leading to Ca^2+^ influx and eNOS recruitment [21,93,296,306,307,308,309]. Furthermore, human brain endothelial cells express NMDA receptors, which interact with group 1 metabotropic glutamate receptors (mGluRs), specifically mGluR1 and mGluR5, to stimulate eNOS and induce NO release [310,311]. Therefore, future studies should aim at determining whether eNOS also supports the peak NVC in humans as well as the maintenance of resting CBF. Consistently, SOCE has been shown to be constitutively active in human cerebrovascular endothelial cells, thus providing a source of Ca^2+^ to sustain basal eNOS activity [183]. Intriguingly, a preliminary investigation reported that amyloid-*β*_(1-40)_ strongly decreases NMDA receptor signaling in mouse brain microvascular endothelial cells [308], suggesting that the endothelial ion signaling machinery may be compromised in neurodegenerative disorders [309,312].

It remains unclear whether human brain capillary endothelial cells can initiate and electrotonically propagate hyperpolarizing signals to upstream arterioles in response to neuronal activity, as reported in the mouse microcirculation (Section 2.1 and Figure 3). However, a combination of transcriptomic analysis and planar patch-clamp recordings has revealed that human brain microvascular endothelial cells express inwardly rectifying K_IR_2.1 channels, K_ATP_ channels, IK_Ca_ channels, and several Ca^2+^-activated Cl^−^ channels, including TMEM16A [201,313]. These findings suggest that EDH may contribute to the portion of the hemodynamic response to sensory stimulation that is resistant to combined nNOS/eNOS inhibition (Figure 7A).

### 3.4. Exploring the Role of Human Endothelial Ion Channels in Ex Vivo Studies

The role of endothelial ion channels in the regulation of human microcirculation has been mainly investigated in ex vivo vessels, particularly when the microvascular bed is located in organs not readily accessible to in vivo studies, such as the heart and adipose tissue.

Briefly, endothelial SK_Ca_/IK_Ca_ channels are primarily responsible for coronary artery vasodilation mediated by EDH in the human cardiac microcirculation (Table 3) [314,315,316]. A recent investigation confirmed that bradykinin elicits intracellular Ca^2+^ oscillations in human coronary microvascular endothelial cells, followed by the recruitment of SK_Ca_/IK_Ca_ channels and the electrotonic propagation of endothelial hyperpolarization to upstream small arteries [61]. Importantly, SK_Ca_ channels are impaired and contribute to microvascular dysfunction in a variety of cardiovascular disorders, including metabolic syndrome [317], diabetes [318,319], cardioplegic ischemia and ischemia–reperfusion injury [288], and cardiopulmonary bypass [315]. Accordingly, pharmacological stimulation of SK_Ca_/IK_Ca_ channels with the specific agonist NS309 [313,320] protected the human coronary microvasculature from cardioplegia-induced hypoxia and reoxygenation injury in diabetic subjects [321]. Conversely, intracoronary infusion of glibenclamide demonstrated that K_ATP_ channels account for ~17% of resting coronary blood flow, while they are not necessary for adenosine-induced vasodilation in human subjects [322]. Nevertheless, a recent investigation reported that adenosine, which can be released in response to an increase in myocardial metabolism [323], elicited EDH by activating endothelial K_ATP_ and K_IR_ channels in ex vivo coronary arterioles (Table 3) [61]. Future studies are, therefore, required to elucidate the role of K_ATP_ channels in the regulation of coronary flow reserve [324,325]. Importantly, genetic susceptibility to coronary microvascular dysfunction and ischemic heart disease has recently been associated with single nucleotide polymorphisms (SNP) in the genes encoding eNOS and the K_IR_6.2 subunit of K_ATP_ channels [326,327]. Current evidence suggests that endothelium-derived NO plays a minor role in the fine regulation of coronary vascular resistance, while it is crucial for flow-mediated vasodilation of upstream small coronary arteries [325,328,329,330]. Furthermore, TRPV4 channels are present in the human coronary microcirculation [331], while the expression of Piezo1 channels has yet to be established. Intriguingly, endothelial-specific deletion of Piezo1 channels reduced capillary density in skeletal muscle but not in cardiac muscle [266], suggesting that Piezo1 channels are not critical for the myocardial vasculature [332].

Adipose arterioles obtained from discarded surgical samples represent a suitable ex vivo model to investigate vascular reactivity in human vessels. A series of studies demonstrated that acetylcholine-induced vasodilation in human adipose tissue from several sources (e.g., pericardial, visceral, and subcutaneous) is mediated by NO and hydrogen peroxide (H_2_O_2_), which in turn elicit VSMC hyperpolarization by activating voltage-gated K^+^ channels and large-conductance Ca^2+^-activated K^+^ channels [331,333,334]. Acetylcholine-induced production of H_2_O_2_ is mediated by NADPH oxidase 4 (NOX4) [333], which mainly releases H_2_O_2_ rather than the superoxide anion (O_2•_**^−^**) [135]. Additionally, acetylcholine-dependent VSMC hyperpolarization may also involve an as-yet-unidentified EDH mechanism, potentially including the recruitment of endothelial SK_Ca_/IK_Ca_ channels driven by the accompanying increase in [Ca^2+^]_i_ (Table 3) [333]. Consistently, acetylcholine-dependent NOX4 activation leads to the activation of TRPV4 channels [331], which are also ROS-sensitive [274,335] and are coupled to SK_Ca_/IK_Ca_ channels [100,122,123]. Flow-induced vasodilation in adipose arterioles also requires the production of H_2_O_2_ and NO [334,336], suggesting that acetylcholine released from endothelial cells facilitates the hemodynamic response to flow [337]. A recent report suggested that flow-dependent vasodilation in adipose arterioles is sustained by K_IR_2.1 channels (Table 3) [338], further implying the upstream recruitment of SK_Ca_/IK_Ca_ channels. Importantly, the vasodilatory response to acetylcholine and flow is impaired in both morbid obesity and coronary artery disease (CAD), conditions that were also associated with a switch from NO- to H_2_O_2_-dependent vasodilation [331,334,339]. Additionally, CAD is associated with increased expression of NOX2, which may replace NOX4 in acetylcholine-induced H_2_O_2_ generation [333]. In line with the impairment of endothelial ion signaling in cardiovascular disorders, endothelial K_IR_2.1 channels are down-regulated in adipose arterioles from hypertensive adults [338].

**Table 3:** The role of endothelial ion channels in the regulation of human coronary and adipose microcirculation.

Ion Channel	Vascular Bed	Vasorelaxing Signaling Pathway	Role	References
SK_Ca_/IK_Ca_ channels	Coronary microcirculation	EDH	Yes	[314,315,316]
SK_Ca_/IK_Ca_ channels	Adipose microcirculation	EDH	Yes	[331,333,334]
K_IR_2.1 channels	Coronary microcirculation	EDH	Yes	[61]
K_IR_2.1 channels	Adipose microcirculation	EDH	Yes	[338]
K_ATP_ channels	Coronary microcirculation	EDH	Yes	[61]
TRPV4 channels	Adipose and coronary microcirculations	EDH	Yes	[331]

Abbreviation: EDH: endothelium-dependent hyperpolarization.

### 3.5. Human In Vivo Evidence, Interindividual Variability, and Translational Limitations

In the previous paragraphs, we have summarized functional evidence from selected human microvascular beds demonstrating the contribution of endothelial ion channels to the fine-tuning of local perfusion. However, in vivo human studies rely on indirect physiological approaches, such as LSD, LSCI, forearm venous occlusion plethysmography, brachial artery flow-mediated dilation (FMD), transcranial Doppler ultrasound and functional magnetic resonance imaging (fMRI). These techniques assess global or integrated indices of endothelial function and infer the role of ion channels through pharmacological manipulation using specific channel blockers, agonists, or openers. As a consequence, they do not allow for the direct assessment of endothelial channel activity at the cellular or molecular level. Furthermore, inter-individual variability represents a major challenge for clinical translation, as genetic background, sex, age, cardiometabolic status, and the inflammatory milieu can markedly influence endothelial channel expression and function, resulting in heterogeneous and context-dependent vascular responses. Additional limitations include the restricted access to human microvascular tissue, the difficulty in controlling systemic confounders in vivo, and the frequent extrapolation from animal models despite known species-specific differences in endothelial signaling pathways. For instance, EDH is critical for NVC in the mouse brain microcirculation [57], whereas NO appears to be the predominant vasorelaxing mediator in the human cerebral microvasculature [296]. These considerations underscore the need for integrative and translational strategies that combine functional human studies with molecular, electrophysiological, and single-cell profiling approaches to enhance the clinical relevance of endothelial ion channel research.

## 4. Future Research Directions

Assessing how endothelial ion signaling fine-tunes human microvascular activity is hampered by intrinsic technical challenges related to interrogating human microvascular function in vivo. Recent advances in translational imaging of human microcirculation may enable the monitoring of functional changes induced by the manipulation of endothelial ion channels directly at or close to the microvascular scale. The commercially available imaging modalities include: positron emission tomography (PET), near-infrared spectroscopy (NIRS), optical imaging, ultrasound imaging, and computed tomography [340]. Additionally, widefield hyperspectral imaging has recently been designed to collect data from a wide field of view, thereby remarkably expanding our ability to investigate human microvascular networks in vivo [341]. It should, however, be pointed out that these novel imaging modalities are mainly suitable for comparing functional changes in microvascular function between healthy subjects and individuals suffering from endothelial dysfunction, which can manifest as cardiovascular disorders [342] or vascular cognitive impairment [309]. By contrast, the topical application of specific drugs to manipulate endothelial ion signaling is restricted by ethical constraints and technical limitations to the periphery, including the skin, limb vasculature, retina, and mucosal tissues [148]. For instance, ethical and technical limitations substantially constrain the investigation of endothelial ion channels in the human cerebral microcirculation. Invasive pharmacological manipulation is strictly restricted by ethical considerations, while the limited accessibility of cerebral microvessels and the lack of endothelial-specific readouts in vivo hinder direct mechanistic assessment. These constraints underscore the need for alternative, minimally invasive experimental approaches with improved cellular resolution.

These hurdles can be circumvented through direct measurements of microvascular function in human tissue samples. We believe that a closer interaction between vascular physiologists and clinicians could be instrumental in increasing the availability of excised pressurized arterioles from a wider range of vascular beds [148]. The classical cannulated approach still represents the most suitable strategy to investigate the physiological role of endothelial ion channels in microcirculation through pharmacological or genetic manipulation [343]. Recently, organoid models derived from human pluripotent stem cells (hPSCs) have been developed to more effectively recapitulate tissue-level complexity; however, the absence of perfusion limits vascular maturation and functional integration. To overcome these limitations, the coupling of organoid systems with microfluidic platforms, such as organ-on-a-chip technologies, is increasingly being explored. The establishment of human microvasculature-on-a-chip platforms incorporating primary endothelial cells, pericytes, and physiologically relevant flow conditions represents a promising tool for mechanistic studies and high-throughput drug screening. Such systems may provide a robust preclinical bridge between reductionist in vitro models and complex human in vivo studies, accelerating the translation of endothelial channel modulation into clinically viable therapies [344,345]. In this context, the application of multi-omics technologies, such as single-cell transcriptomics, proteomics, and spatial metabolomics, will be instrumental in generating comprehensive maps of ion channel expression and regulation within human microcirculatory endothelial cells under physiological and pathological conditions. These datasets could uncover previously unrecognized channel networks and patient-specific signatures relevant to disease progression and therapeutic responsiveness [346,347].

In line with this, animal studies have shown that endothelial ion channel activity can be severely compromised by endothelial dysfunction, thereby contributing to multiple cardiovascular disorders, including hypertension, type 2 diabetes, metabolic syndrome, hypercholesterolemia, vascular cognitive impairment, and aging, and, more recently, to viral infections and brain disorders, such as Alzheimer’s disease and traumatic brain injury. A detailed discussion of the mechanisms underlying the disruption of the endothelial ion signaling machinery falls beyond the scope of the present article, which is focused on describing the physiological role of endothelial ion channels in the control of human microcirculation. We refer to the reader to several recent review articles that exhaustively cover this aspect of endothelial pathology [34,135,348,349,350,351,352,353,354]. Herein, we would like to point out that assessing the specific role of endothelial ion channels in the regulation of local blood flow is likely to highlight novel pharmacological targets for the treatment of cardiovascular and neurological disorders. Studies conducted in animal models suggest that the pharmacological activation of endothelial SK_Ca_/IK_Ca_ channels with the selective agonist SKA-31 stimulates vasodilation in the presence of atherosclerosis [355], type 2 diabetes [356], and aging [357]. Unfortunately, SKA-31 induces undesired side effects, such as heart rate reduction and central sedation, and is unlikely to enter clinical investigations [198]. K_IR_2.1 channels also support EDH. K_IR_2.1 channel activity absolutely depends on binding of PIP_2_ to the channel [56]. Systemic administration of a PIP_2_ analogue was recently found to restore NVC in mouse models of somatosensory stimulation [358] and small vessel disease (SVD) [359]. Consistently, a recent investigation showed that GSK1059615, which inhibits phosphatidylinositol-3-kinase (PI3K) and thereby increases the bioavailability of PIP_2_, enhanced endothelial K_IR_2.1 currents and the hemodynamic response to somatosensory stimulation in mice affected by SVD [360]. These findings support the conclusion that targeting the K_IR_2.1 channel may be a viable therapeutic strategy to treat endothelial dysfunction. Five classes of PI3K inhibitors have been approved by the Food and Drug Administration (FDA) for cancer treatment, including alpelisib [361], which selectively targets the endothelial PI3Kα isoform [362]. Several clinical studies have evaluated the therapeutic efficacy of alpelisib in patients with vascular malformations, but the duration of the optimal therapy and the potential for resistance warrant future investigations [363]. It is, however, still unknown whether PI3K inhibition stimulates endothelial K_IR_2.1 channel activity in humans. Targeting TRP channels may also provide a promising therapeutic strategy to restore microvascular function. For instance, systemic TRPV1 activation can reduce MAP in hypertension [364] and promote therapeutic angiogenesis in ischemic disorders [117]. Several pharmacological approaches aim to increase TRPV1 activation, including the dietary agonist capsaicin, the natural agonist Resiniferatoxin, and an increasing number of synthetic drugs, including the lipid-lowering drugs simvastatin and levodiamine, N-arachidonoyldopamine (NADA) and N-oleoyldopamine (OLDA) [117,365]. Additional TRPV1 agonists, such as capsaicin-gel, capsaicin liquid, capsaicin patch, CNTX-4975, CA-008, Zostrix, RTX (Lopain, MTX-071), have been designed to alleviate neuropathic pain disorders and are under clinical trial. It is, however, still unclear whether they may also be beneficial in stimulating microvascular activity in cardiovascular disorders [366]. We have recently suggested that stimulation of endothelial TRPV1 channels can be achieved by combining optical excitation and organic polymer excitation [112,367,368], as detailed in [369,370]. TRPV4 channels can also be modulated by multiple dietary, natural, and synthetic agonists [275], such as GSK1016790A, apigenin, eugenol, curcumin, omega-3 fatty acid, and 4α-PDD (4α-Phorbol 12,13-didecanoate). It has been proposed that targeting endothelial TRPV4 channels could be instrumental in treating many cardiovascular disorders, including hypertension, atherosclerosis, ischemic and metabolic disorders, and viral infections [354,371,372,373,374]. Finally, it has been suggested that Piezo1 agonists, such as Yoda1 and Yoda2, may be beneficial for the treatment of both cardiovascular and neurodegenerative disorders, including systemic hypertension, pulmonary hypertension, and Alzheimer’s disease [375,376]. Nevertheless, the pharmacological targeting of endothelial TRPV4 or Piezo1 channels is still far from translational applications.

## 5. Conclusions

Endothelial ion channels are emerging as key regulators of human microcirculatory function. While unveiling the contribution of specific endothelial ion channels to local blood flow regulation in human subjects is hampered by technical difficulties and ethical constraints, these studies remain essential for translating findings from in vitro endothelial models or ex vivo isolated vessels to the in vivo clinical setting. Our analysis suggests that a distinct repertoire of endothelial ion channels engages heterogenous endothelium-dependent vasorelaxant pathways across different vascular beds. For instance, cutaneous vasodilation in response to heat stress is primarily driven by Ca^2+^-dependent production of NO and PGI2, while K_ATP_ channels may serve as a backup vasorelaxant pathway that replaces NO signaling when SK_Ca_/IK_Ca_ channels are impaired. Conversely, EDH mediated by SK_Ca_/IK_Ca_ and K_IR_2.1 channels plays a central role in functional sympatholysis and exercise hyperemia in skeletal muscle. This body of preliminary evidence is consistent with the well-recognized functional heterogeneity of endothelial signaling along the systemic vasculature and warrants further validation by future studies addressing several unresolved issues.

First, there is currently no direct evidence that the EDH model established in animal models, whereby endothelial hyperpolarization is primarily sustained by SK_Ca_/IK_Ca_ channels in arterioles (Figure 3) and K_IR_/K_ATP_ channels (Figure 4) in capillaries, can be fully translated to the human microcirculation. Second, while NO supports the cutaneous vasodilatory response elicited by acetylcholine and likely histamine, the specific G_q_PCR subtypes involved and the underlying Ca^2+^ signaling mechanisms remain poorly defined. Similarly, it has yet to be proven that ATP contributes to functional sympatholysis and, if so, through which purinergic receptor subtype in skeletal muscle. Third, indirect evidence suggests that eNOS drives NVC in the human brain microcirculation, but this hypothesis must be directly tested using selective eNOS inhibitors. The role of endothelial glutamate receptors also deserves further investigation, as they are plausible candidates for translating somatosensory stimulation into an NVC response. Fourth, the mechanosensitive ion channels that support NO production following the initial rise in local blood flow remain to be identified. Piezo1 and TRPV4 channels are proposed to serve as shear stress sensors in human microcirculation, but this concept awaits experimental confirmation. Fifth, future studies are required to assess which thermosensitive TRP channels contribute to active vasodilation in the skin and skeletal muscle. Emerging evidence supports a role for TRPV1, whereas the contribution of the polymodal TRPV4 channel warrants further investigation.

The involvement of these ion channels in the progression of microvascular dysfunction—a common denominator in cardiovascular disorders—as well as in neurodegenerative pathologies, such as Alzheimer’s disease, is only beginning to emerge. Animal studies, together with the preliminary findings in human subjects discussed throughout this manuscript, suggest that the disruption of the endothelial ion signaling machinery may exacerbate endothelial injury. Elucidating the specific contribution of human endothelial ion channels to different microvascular beds opens novel therapeutic perspectives. Targeting these channels could provide innovative strategies to restore endothelial function and improve tissue perfusion in a wide range of cardiovascular and metabolic diseases, supporting a shift towards more precise, channel-based vascular medicine.

## Data Availability

No new data were created or analyzed in this study. Data sharing is not applicable to this article.
